# Clinical, cognitive and psychopathological markers of suicide attempts in eating disorders

**DOI:** 10.1192/j.eurpsy.2026.10350

**Published:** 2026-07-31

**Authors:** L. Di Lodovico

**Affiliations:** Clinique des Maladies Mentales et de l’Encéphale, GHU Paris Psychiatrie et Neurosciences, Paris, France

## Abstract

**Abstract:**

Suicide is a major contributor to excess mortality in eating disorders, particularly in anorexia nervosa, and recent research highlights a convergence of clinical severity and biological dysregulation as key contributors to risk.

Across recent longitudinal, ecological, and meta-analytic studies, the most consistent clinical markers include prior suicide attempts, severe depressive symptoms, emotion dysregulation, compulsive or rigid behavioral patterns, low body mass index, chronicity of illness, and high levels of eating-disorder psychopathology, with dynamic fluctuations in negative affect and interpersonal stress closely linked to momentary suicidal ideation.

Cognitive factors predisposing to suicide risk encompass deficits in cognitive flexibility, impaired decision-making abilities, perfectionism, impulsivity, increased rumination, and reduced interoceptive awareness.

Emerging biological findings suggest that suicide risk in eating disorders is associated with alterations in stress-response systems, particularly hypothalamic–pituitary–adrenal axis dysregulation and abnormal cortisol patterns, as well as inflammatory activation (altered cytokines), metabolic and lipid abnormalities related to starvation, and serotonergic and neurotrophic changes (BDNF) affecting mood regulation, impulsivity, and cognitive control.

Integrative models propose that chronic malnutrition interacts with affective vulnerability, compulsivity, and increased pain tolerance to simultaneously heighten suicidal desire and capability, especially in restrictive phenotypes.

Overall, current evidence supports a multidimensional understanding of suicide risk in eating disorders, in which clinical severity, emotional processes, and biological stress and metabolic pathways jointly contribute to vulnerability, underscoring the need for multimodal assessment and prevention strategies. Improved knowledge of the cognitive mechanisms associated with suicide risk in this population could guide the development of tailored treatment and prevention approaches.

**Disclosure of Interest:**

None Declared

